# Commentary: Induction of hepatic fibrosis in mice with schistosomiasis by extracellular microRNA-30 derived from *Schistosoma japonicum* eggs

**DOI:** 10.3389/fimmu.2025.1604905

**Published:** 2025-06-09

**Authors:** Haoran Zhong, Yamei Jin

**Affiliations:** National Reference Laboratory for Animal Schistosomiasis, Key Laboratory of Animal Parasitology of Ministry of Agriculture and Rural Affairs, Shanghai Veterinary Research Institute, Chinese Academy of Agricultural Sciences, Shanghai, China

**Keywords:** Schistosomiasis, *Schistosoma japonicum*, liver fibrosis, extracellular vesicle, miRNA

## Introduction

Schistosomiasis, a parasitic disease caused by *Schistosoma* species, poses a significant public health threat, particularly in tropical and subtropical regions (1). The primary consequence of chronic infection with *Schistosoma japonicum* and *Schistosoma mansoni* is the development of liver fibrosis, driven largely by the host’s immune response to eggs trapped in the liver (2). Upon exposure to soluble egg antigens (SEAs), hepatic stellate cells (HSCs) become activated and begin to secrete multiple profibrotic chemokines, disrupting the balance of extracellular matrix (ECM) remodeling. This leads to the development of granulomas and hepatic fibrosis, with HSCs playing a central role in coordinating the immune response and facilitating collagen deposition around trapped schistosome eggs (3). Recent research indicates that extracellular vesicles (EVs) derived from *S. japonicum* eggs deliver microRNAs (miRNA), such as sja-miR-71a and sja-miR-2162, which activate the host’s TGF-β/Smad signaling pathway, thereby regulating HSC activation and fibrosis (4–7). Despite these advancements, the complex mechanisms of long-term interactions between *Schistosoma* and the host remain poorly understood. Ongoing research into these processes is crucial for uncovering new insights into host-parasite dynamics and identifying potential therapeutic targets to mitigate liver fibrosis.

## General comments

In a recent study entitled “Induction of hepatic fibrosis in mice with schistosomiasis by extracellular microRNA-30 derived from *Schistosoma japonicum* eggs” was published in Frontiers in Immunology (8). Chen et al. investigated the role of miRNA-30 derived from *S. japonicum* egg EVs in the induction of hepatic fibrosis in mice. Through *in vitro* experiments, they demonstrated that the miRNA-30 activates HSCs and promotes the expression of key fibrotic markers. Using recombinant adeno-associated virus (rAAV) vectors, the authors further assessed the *in vivo* effects of the miRNA-30 overexpression and inhibition. They found that overexpression of the miRNA-30 in infected mice exacerbated hepatic fibrosis, while silencing the miRNA-30 alleviated the symptoms, as evidenced by reduced collagen deposition and smaller granuloma size. The study highlights miRNA-30 as a crucial factor in schistosome-induced hepatic fibrosis and suggests it may serve as a potential therapeutic target for mitigating liver damage in schistosomiasis. We greatly appreciate this work and acknowledge its significant contribution. However, it may be beneficial to further refine the experimental design details is needed in the research on the role of *S. japonicum* EVs in host liver fibrosis.

First, in terms of description, the use of the term “exosome” is not entirely accurate, and a more appropriate term would be “extracellular vesicles” (EVs). Generally, the term “exosome” should be strictly reserved for vesicles of endosomal origin that are released through specific exocytosis pathways (9). In the case of helminths, very few studies have focused on understanding the origin of different vesicles, and this study does not provide evidence for the endocytic origin of *S. japonicum* egg-derived EVs. Therefore, it may be helpful for the authors consider following the recently published guidelines for the study of helminth-derived EVs (10).

Secondly, two key aspects regarding the methods may require refinement to meet the current standards for helminth EVs research and schistosomiasis study design. Based on the aforementioned reference (10), it would be valuable for researchers to provide more detailed information on the EV extraction process. Specifically, it would be helpful for the authors to systematically supplement the following technical parameters: a comprehensive description of the *S. japonicum* egg separation protocol, including enzymatic/mechanical disruption schemes, filtration parameters (pore size, filter membrane type), and centrifugation conditions (duration, centrifugal force, temperature control); clarification of biological material quantification details (the total number of eggs used for EV collection per experiment should be ≥3 biological replicates); a complete breakdown of the culture medium components (type of base medium, serum source/concentration, antibiotic regimen, EV removal method), and the culture conditions (duration, gas environment, temperature); and refinement of the key parameters in the EV enrichment protocol, such as ultracentrifugation (rotor model, k-factor, sedimentation path) and quality control measures (calibration details for nanoparticle tracking analysis, protein quantification methods). Providing these details would significantly enhance the reproducibility of the experiments and offer a more robust foundation for future studies. Additionally, in the *in vivo* experiments, liver egg counts were used to assess whether the effect of rAAV is directed toward granulomas rather than the egg-laying capacity of the parasite. While this design is important, further clarification on the specific methodology employed would be beneficial. Based on the egg count data (evidenced by 4,000-6,000 egg counts), it seems that only a portion of the liver was collected. Given the uncertainty surrounding potential egg retention at various sites due to their flow through the bloodstream, sampling only part of the liver may introduce inaccuracies (11). Therefore, it may be more appropriate to sample the entire liver tissue for analysis.

Finally, regarding future directions for related research, the question arises as to whether the miRNA described in the study is exclusively derived from the eggs. Could the adult worms also secrete this miRNA? If the adult worms are indeed capable of secreting this miRNA, the pathological changes induced by this miRNA may also be attributed to the worms themselves (12). If, as suggested by the authors, this miRNA is indeed derived from the eggs, it would be beneficial to add experimental groups that include egg-derived EVs depleted of miRNA and another group with egg-derived EV-depleted products to further confirm the role of this miRNA in the egg-derived EVs (10).

## Discussion

Overall, we highly appreciate the work presented in this study, which effectively demonstrates the role of miRNA-30 derived from *S. japonicum* egg EVs in inducing hepatic fibrosis in mice. The findings offer valuable insights into potential interactive mechanisms and therapeutic targets for schistosomiasis-related liver damage. However, further refinement of the experimental details and additional supporting information could significantly advance the field of helminth EV research and enhance the reproducibility and impact of future studies.
